# Orientin Enhances Colistin-Mediated Bacterial Lethality through Oxidative Stress Involvement

**DOI:** 10.1155/2022/3809232

**Published:** 2022-05-09

**Authors:** Madonsela Khumbulani, Kazeem Adekunle Alayande, Saheed Sabiu

**Affiliations:** ^1^Department of Biotechnology and Food Science, Faculty of Applied Sciences, Durban University of Technology, Durban, South Africa; ^2^Unit for Environmental Sciences and Management, North-West University, Potchefstroom, South Africa

## Abstract

Bacterial resistance to colistin has prompted the search for alternative strategies to enhance antibacterial potential. Combination therapy remains one of the viable strategies in antibacterial therapy and has been proven to be effective in reducing the risk of resistance. In this study, the potential of orientin for enhancing the antibacterial activity of colistin was assessed against *Klebsiella pneumoniae* and *Pseudomonas aeruginosa in vitro*. The involvement of oxidative stress in such enhancement was also assessed. The minimum inhibitory concentrations (MICs) of colistin and orientin were 16 *μ*g/mL and 64 *μ*g/mL against *K. pneumoniae* and 64 *μ*g/mL and 256 *μ*g/mL against *P. aeruginosa* respectively. For the combination therapy, orientin potentiates the antibacterial effect of colistin with a friction inhibitory concentration index (FICI) of 0.37 and 0.31 against *K. pneumoniae* and *P. aeruginosa*, respectively. This observation suggests a synergistic interaction, with the MIC of colistin being reduced by 3- and 4-fold in the presence of orientin against *K. pneumoniae* and *P. aeruginosa*, respectively. Additionally, treatment with the combination of colistin and orientin induced oxidative stress against both organisms through increased cellular levels of superoxide anion radicals with concomitant increase in NAD^+^/NADH and ADP/ATP ratios. These findings suggest that orientin enhanced colistin in the killing of the test bacteria and the cotreatment of colistin and orientin induced oxidative stress, through reactive oxygen species generation, which consequently facilitated bacterial lethality without causing drug-drug interactions. Although, the data presented in this study has supported the capability of orientin for strengthening antibacterial activity of colistin toward the fight against drug-resistant Gram-negative bacteria, studies focusing on the exact target and mechanism of action of orientin are underway.

## 1. Introduction

Antibiotic resistance remains a persistent health challenge, claiming more than 750000 deaths each year and this has been largely attributed to the overuse and misuse of antibiotics coupled with bacterial evolution [1]. Some bacteria resist antibiotics by altering their genetic material and thus forming antibiotic-resistant genes [2]. While antibiotics such as glycopeptides, aminoglycosides, macrolides, and derivatives through chemical modification of existing antibiotics have been used as improved alternatives in overcoming bacterial resistance to antibiotics, available evidence suggests that adverse effect, continuous resistance evolution, and cost have undermined their application, hence presenting them as less effective [1]. Even colistin, which has been used as a last resort whenever the use of aminoglycosides, quinolones, and *β*-lactams is not effective [3], has now been reported to be less potent due to resistance from several bacterial strains [4].

Over the years, plant secondary metabolites have gained research interest due their diverse pharmacological properties including antibacterial, antiviral, antifungal, antioxidant, and anti-inflammatory activities [5]. Specifically, both Gram-negative and Gram-positive bacterial strains have shown susceptibility to several isolated compounds from plants including phenolics [6]. Studies have also implicated phenolics in antibacterial combination therapy with conventional antibiotics, and such combination therapy has been reported to enhance the antibacterial potential of antibiotics [7, 8].

Antimicrobial combination therapy is one of the viable strategies in clinical practice and has been used to enhance therapeutic action of antibiotics against multidrug-resistant bacterial strains of several infectious diseases [9], to mitigate toxicity [10], and to prevent the emergence of drug resistance [11, 12]. Antibiotics can be used in combination with other antibiotics or with other antibacterial agents. For instance, colistin has been reported to exhibit synergistic action against *Acinetobacter baumannii* when combined with phenolic acids. Besides enhancing the bacterial lethality of conventional antibiotics, phenolic acids such as gallic acid, caffeic acid, and protocatechuic acid with a catechol functional group have been found to act as redox cycler in a manner that generates reactive oxygen species (ROS) and semiquinone [13]. The ROS generated in the process have been documented to contribute toward bacterial killing *in vitro* [13].

**Figure 1:**
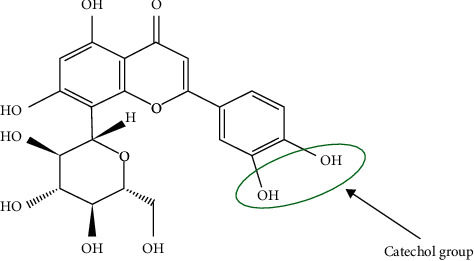
Structure of orientin (https://pubmed.ncbi.nlm.nih.gov/).

Among the *C*-glycosylated flavonoids, orientin, isoorientin, vitexin, and isovitexin are the most frequently implicated therapeutics due to their high stability [14], with orientin finding significant antibacterial applications against *Staphylococcus aureus*, *Escherichia coli*, and *Bacillus subtilis* [14, 15]. Additionally, orientin has a catechol functional group (Figure 1) and may generate ROS as a by-product during catechol oxidation. It is therefore hypothesized that the use of orientin in combination therapy with colistin could enhance the effectiveness of colistin against the ever-increasing infectious diseases caused by pathogenic bacteria that are becoming more difficult to treat. Hence, in this study, the ability of orientin to potentiate colistin against multidrug-resistant Gram-negative bacteria (*Klebsiella pneumoniae* and *Pseudomonas aeruginosa*) was evaluated *in vitro*. The involvement of ROS in such enhancement through monitoring of some important oxidative stress biomarkers was also investigated, while the tendency of the combination of colistin and orientin to cause drug-drug interaction was established *in silico*.

## 2. Materials and Methods

### 2.1. Bacterial Cultures, Antibiotics, and Test Compounds

The *Klebsiella pneumoniae* and *Pseudomonas aeruginosa* strains used in this study were obtained from Anatech Analytical Technology, Olivedale, Gauteng, South Africa. Colistin and orientin were procured from Merck, South Africa, and their stock solutions were prepared by weighing and subsequent dissolution in sterile distilled water. The resulting stock solutions were then preserved at 4°C until further use.

### 2.2. *In Vitro* Antibacterial Evaluation

Before the determination of minimum inhibitory concentration (MIC), agar well diffusion assay was employed [16], to test whether *K. pneumonia* and *P. aeruginosa* were susceptible to colistin and orientin. Briefly, the surface of the agar plates was inoculated with 100 *μ*L of the exponential phase microbial inoculum (*K. pneumonia* and *P. aeruginosa*). Following inoculation, a hole was bored on the surface of the plate with the tip of a sterile cork borer. Thereafter, 50 *μ*L of varying concentrations of colistin and orientin solution was then introduced into the well. The plates were then incubated at 37°C for 24 h. Thereafter, the broth microdilution assay for MIC determination was performed as earlier reported [16], following the clinical and laboratory standard institute guidelines [17] for both orientin and colistin. This was then followed by the evaluation of the minimum bactericidal concentrations (MBCs) against *K. pneumoniae* and *P. aeruginosa*. In brief, two-fold serial dilutions of orientin and colistin were prepared for the determination of the MIC. The bacterial suspensions were prepared by transferring colonies aseptically into sterile saline (0.85%). The turbidity was adjusted to 0.5 McFarland standards using spectrophotometer at 600 nm. The prepared orientin (1024 *μ*g/mL) and colistin (512 *μ*g/mL) solutions were mixed with the bacterial suspension in a 96-well microtiter plate. After adequate mixing, the microtiter plate was then incubated at 37°C for 24 h. The experiment was done in triplicate, and the MICs in the two organisms were determined from the well with the lowest concentration showing no turbidity bacterial growth. For the MBC determination, on the other hand, nutrient agar plates were used for plating the dilutions representing at least two concentrations of colistin and orientin above the MIC, followed by incubation for 48 h at 37°C, and the colonies were counted to determine the viable CFU/mL.

### 2.3. Checkerboard Assay

For the determination of the combined antibacterial effect of orientin with colistin against *K. pneumoniae* and *P. aeruginosa*, checkerboard assay was employed as previously described [18]. Using the prepared stock solution of orientin and colistin, two-fold serial dilutions were performed in Mueller–Hinton broth (MHB) in sterile 96-well microtiter plates in decreasing concentrations. The standardized (0.5 McFarland) suspensions of *K. pneumoniae* and *P. aeruginosa* were inoculated into the wells of the microtiter plates. The wells containing the broth but no bacteria served as negative controls, and the ones with both broth and suspension of either *K. pneumoniae* or *P. aeruginosa* suspensions represented positive controls. The friction inhibitory concentration index (FICI) was then calculated to evaluate the combined effect of colistin and orientin using the following formula:

(1) $$\begin{array}{c} \mathrm{FICI}=\frac{\mathrm{MIC}\;\mathrm{of}\;\mathrm{orientin}\;\mathrm{in}\;\mathrm{combination}\;\mathrm{with}\;\mathrm{colistin}}{\mathrm{MIC}\;\mathrm{of}\;\mathrm{orientin}\;\mathrm{alone}}+\frac{\mathrm{MIC}\;\mathrm{of}\;\mathrm{colistin}\;\mathrm{in}\;\mathrm{combination}\;\mathrm{with}\;\mathrm{orientin}}{\mathrm{MIC}\;\mathrm{of}\;\mathrm{colistin}\;\mathrm{alone}}. \end{array}$$

The probable combined effect of colistin and orientin was established in accordance with an earlier method [19], where an FICI of 0.5 or below signifies synergism, values above 0.5 and less than 4 indicate indifferent interaction, and values above 4.0 indicate antagonistic effect.

### 2.4. Time-Kill Susceptibility Test

The rate at which treatment with either colistin or orientin and their combination kills bacteria (*K. pneumoniae* and *P. aeruginosa*) over time was evaluated [13, 20]. Using a 96-well microtiter plate, 50 *μ*L of MHB was added in each well followed by the addition of colistin, orientin, and combination of colistin with orientin. Following the addition of antimicrobial agents, 50 *μ*L of the standard inoculum of *K. pneumoniae* and *P. aeruginosa* was inoculated into the wells of the microtiter plates. The growth control wells were comprised of only MHB and bacterial inoculum. Thereafter, the microtiter plates were incubated at 37°C to measure the optical density at 0, 2, 4, 6, 8, and 24 h after the addition of bacterial inoculum. The time-kill curves were plotted as the decrease in the optical density within the experimentation period.

### 2.5. Oxidative Stress Biomarker Assays

#### 2.5.1. NAD^+^/NADH Assay

The ratio of NAD^+^/NADH in *K. pneumoniae* and *P. aeruginosa* cells treated with colistin, orientin, and their combination was estimated based on the procedure outlined in NAD^+^/NADH quantification kit (Sigma-Aldrich, MAK 037). The bacterial cells at exponential phase were incubated with colistin (with or without orientin) and with orientin alone at a resulting concentration of 4 × MIC for each treatment for 30 minutes at 37°C. After incubation, cold phosphate buffer saline (pH 7.5) was utilized to wash bacterial cells, followed by centrifugation (2000 ×g, 5 minutes). Treated cells were frozen/thawed for two cycles on dry ice for 20 minutes followed by 10 minutes at room temperature using NAD^+^/NADH extraction buffer. Thereafter, samples were centrifugated (13000 ×g, 10 minutes) to separate the cell-free extract. Subsequently, NAD^+^/NADH extraction buffer (50 *μ*L) was used to treat 50 *μ*L of the cells and then followed by the addition of 100 *μ*L of master mix (NAD cycling buffer and NAD cycling enzyme). After complete mixing, the reaction was incubated for 5 minutes at room temperature. Thereafter, the absorbance was read at 450 nm following the addition of NADH developer (10 *μ*L) and incubation for 2 h at room temperature. The ratio of NAD^+^/NADH in the samples was then determined using the following equation:

(2) $$\begin{array}{c} \frac{{\text{NAD}}^{+}}{\text{NADH ratio}}=\frac{\text{NADtotal}-\text{NADH}}{\text{NADH}}, \end{array}$$

where NAD^+^_total_ is the amount of total NAD^+^ (NAD^+^ + NADH) in the unknown sample (treated bacterial cells) (pmole) from the standard curve and NADH is the amount of NADH in treated bacterial cells (pmole) from the standard curve.

#### 2.5.2. ADP/ATP Assay

For the determination of ADP/ATP ratio, the procedure outlined in ADP/ATP ratio quantification kit (Sigma-Aldrich, MAK 135) was employed. The *K. pneumoniae* and *P. aeruginosa* cells at exponential phase were treated with colistin (with or without orientin) and with orientin alone (with a resulting concentration of 4 × MIC for each treatment) and incubated for 30 minutes at 37°C. An equal volume of ATP reagent (90 *μ*L) was mixed with the cells and incubated for 1 minute at room temperature. Luminescence (relative light units) was read for ATP assay (RLU_A_). The luminescence for ATP (RLU_B_) was read to provide the background before ADP measurement after the mixture was incubated for 10 minutes. After reading (RLU_B_), ADP reagent (5 *μ*L) was added and mixed immediately, and the luminescence (RLU_C_) was read after 1 minute. Then ADP/ATP ratio was estimated using the following equation:

(3) $$\begin{array}{c} \frac{\mathrm{ADP}}{\mathrm{ATP}\;\mathrm{ratio}}=\frac{{\text{RLU}}_{C}-{\text{RLU}}_{B}}{{\text{RLU}}_{A}}. \end{array}$$

#### 2.5.3. Superoxide Anion Radical Assay

The *K. pneumoniae and P. aeruginosa* cells were grown into exponential phase. Following incubation, the cells were further incubated with 0.5 mL of 4 × MIC of colistin, orientin, and their combination (with a resulting concentration of 4 × MIC) for 30 minutes at 37°C. Thereafter, 0.25 mL of nitroblue tetrazolium (1 mg/ml) was added and incubated for another 30 minutes at 37°C. Following incubation, 0.05 mL of 0.1 mM HCl was added, followed by centrifugation at 1500 ×g for 20 minutes. The nitroblue tetrazolium that was reduced in the pellets was extracted and further diluted with 0.8 mL of phosphate-buffered saline (pH 7.5). A microtiter plate reader absorbance was then used to read the absorbance at 575 nm. Thereafter, the amount of superoxide anion radical generated was calculated using a molar extinction coefficient of 3-(4,5-dimethylthiazol-2y-l)-2,5-diphenyltetrazolium bromide formazan (17000 M^−1^·cm^−1^ at pH 7.4–8) as described by [13] and then converted to percentage.

### 2.6. Evaluation of Probable Drug-Drug Interaction between Colistin and Orientin

For molecular docking, the crystal structure of cytochrome 3A4 (CYP3A4) was obtained from RCSB protein data bank (https://www.rcsb.org/) and used as rigid molecule receptor for colistin, orientin, ketoconazole, and rifampicin. The 3D structures of colistin and rifampicin were obtained from ChemSpider (http://www.chemspider.com/) while those of orientin and ketoconazole were obtained from PubChem in sdf format (https://pubchem.ncbi.nlm.nih.gov/). The nonstandard residues, complexes such as nonessential water molecules, and heteroatoms that were bound to the active site of CYP3A4 were removed using UCSF Chimera 1.15 software to prepare CYP3A4 for docking. Gasteiger charges were added to the molecule, and nonpolar hydrogens atoms were merged into carbon atoms prior to docking. The grid box, with a spacing of 1 Å and size of 31.13 × 30.54 × 26.54 pointing toward *x*, *y*, and *z* directions, was firstly defined to dock the compounds to the binding site of CYP3A4. The binding energies of colistin and orientin were then compared with those of conventional inhibitor (ketoconazole) and inducer (rifampicin) of CYP3A4. Furthermore, SwissADME (http://www.swissadme.ch/) was utilized to predict the ADMET properties of colistin and orientin against CYP3A4.

### 2.7. Statistical Analysis

GraphPad Prism version 5.0 using one-way ANOVA was utilized to analyze the *in vitro* results, followed by nonparametric tests to detect any significant difference (*p* < 0.05) between the treatment means. The results are presented as mean ± standard error of the mean (SEM).

## 3. Results

### 3.1. Antibacterial Activity

The results obtained from the agar well diffusion assay revealed that the test organisms were susceptible to colistin and orientin, with larger zones of inhibition observed against *K. pneumonia* compared to *P. aeruginosa* (Figure S1). The zones of inhibition obtained with colistin (256 ug/mL) were 23 mm and 18 mm against *K. pneumonia* and *P. aeruginosa*, respectively, while they were 11 mm and 25 mm for *P. aeruginosa* and *K. pneumonia*, respectively, following treatment with orientin (512 *μ*g/mL) (Table 1). Furthermore, the data obtained with respect to MICs of the test compounds revealed that colistin and orientin had values less than ≤64 *μ*g/mL with MBC ranging between 128 and 512 *μ*g/mL against *K. pneumoniae*, while they had MICs ≤256 *μ*g/mL with MBC ranging between 256 and 1024 *μ*g/mL against *P. aeruginosa* (Table 1). From the checkerboard assay, it was observed that the combination of colistin and orientin resulted in a synergistic interaction as the FICI was 0.37 and 0.31 against *K. pneumoniae* and *P. aeruginosa*, respectively (Table 1).

### 3.2. Time-Kill Analysis

**Figure 2:**
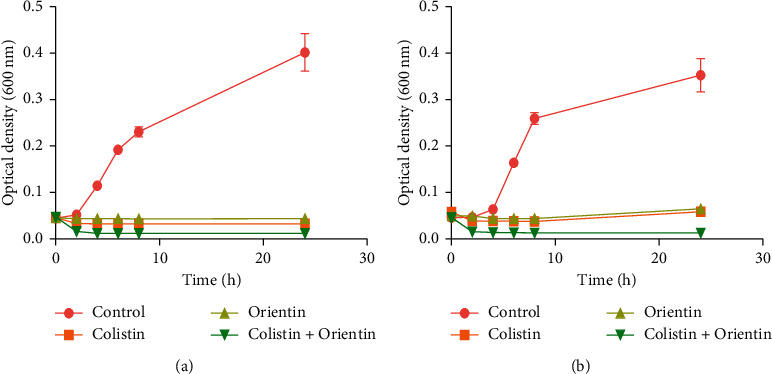
Time-kill growth curves of (a) *K. pneumoniae* and (b) *P. aeruginosa* treated with 4 × MIC of colistin, orientin, and their combination. No significant difference (*p* < 0.05) was observed with colistin and orientin alone.

Treatment with colistin and orientin alone as well as their combination resulted in decreased number of viable bacterial cells measured as the optical density of *K. pneumoniae* which decreased after 2 h of treatment and remained constant throughout the 24 h exposure time (Figure 2(a)). For *P. aeruginosa*, only the combination of colistin and orientin showed an observable reduction in optical density after 2 h of treatment and was maintained over the 24 h exposure period (Figure 2(b)). A noticeable increase in viable cells was however observed in treatments with colistin and orientin alone after 24 h of exposure (Figure 2(b)).

### 3.3. Oxidative Stress Markers

**Figure 3:**
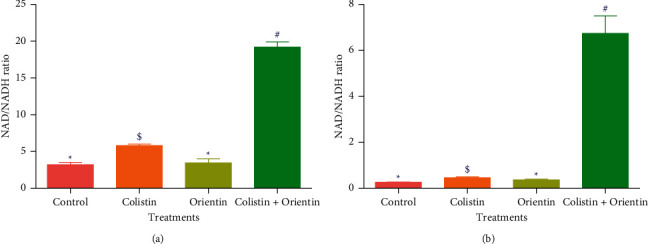
NAD+/NADH ratio in (a) *K. pneumoniae* and (b) *P. aeruginosa* treated with dH_2_O, colistin, orientin, and colistin with orientin (4 × MIC). Bars with different symbols are significantly different (*p* < 0.05) from each other.

**Figure 4:**
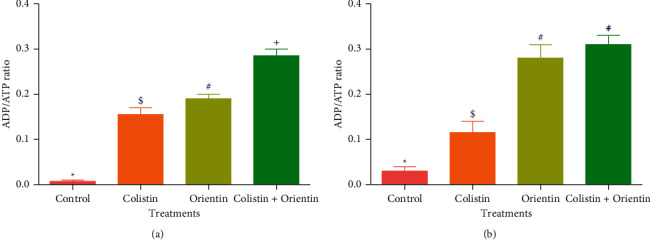
ADP/ATP ratio in (a) *K. pneumoniae* and (b) *P. aeruginosa* cells treated with dH_2_O, colistin, orientin, and colistin with orientin (4 × MIC). Bars with different symbols are significantly different (*p* < 0.05) from each other.

**Figure 5:**
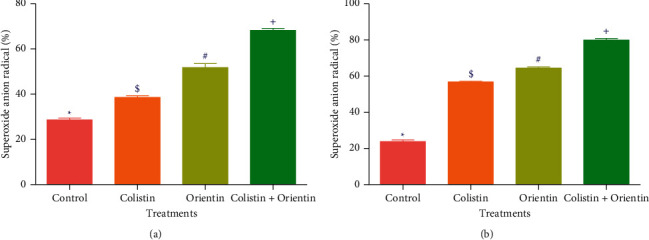
Superoxide anion radical generated following the treatment with colistin, orientin, and combination of colistin with orientin at 4 × MIC against (a) *K. pneumoniae* and (b) *P. aeruginosa*. Bars with different symbols are significantly different (*p* < 0.05).

The results of involvement of ROS/oxidative stress in the bacterial lethality of the test compounds are presented in Figures 3–5. The cellular levels of NAD^+^/NADH and ADP/ATP ratios increased significantly (*p* < 0.05) in both *K. pneumoniae* and *P. aeruginosa* cells following treatment with test compounds relative to cells treated with sterile distilled water (control) (Figures 2–4). The *K. pneumoniae* and *P. aeruginosa* cells treated with colistin and orientin as well as their combination were significantly different (*p* < 0.05) regarding generation of superoxide anion radical compared to cells treated with sterile distilled water, with the combined treatment having the most profound effect in each case (Figures 5(a) and 5(b)).

### 3.4. Drug-Drug Interactions

From the molecular docking results, colistin and orientin had binding energy values of −8.0 kcal/mol and −9.0 kcal/mol with 10 and 5 hydrogen bonds, respectively (Table 2, Figures S2 and S3). These values are more or less the same as those of ketoconazole (−9.5 kcal/mol) and rifampicin (−7.7 kcal/mol), but with 2 and 3 hydrogen bonds, respectively (Table 2, Figures S4 and S5). A further probe into their probable drug-drug interaction tendency with SwissADME predicts both colistin and orientin to be neither inducers nor inhibitors of CYP3A4 (Table 2).

## 4. Discussion

Due to the devastating effect of multidrug-resistant strains of Gram-negative bacteria, the reuse of colistin has been advocated and becoming increasingly embraced [4]. However, despite their efficacy, some Gram-negative bacteria belonging to the Enterobacteriaceae family such as *K. pneumoniae* and *P. aeruginosa*, which are implicated in diseases such as bacteremia, septicemia, hospital acquired pneumonia and lung infections, and ventilator-associated pneumonia, have remained consistently resistant to colistin [3]. Since colistin is regarded as the last hope for treating infections caused by Gram-negative bacteria, strategies to overcome this menace are highly needed. Among the available strategies, combination therapy has been recognized as one of the viable options in enhancing the antibacterial potency of antibiotics [9]. Previously, it has also been reported that phenolics with catechol functional group such as protocatechuic acid, ferulic acid, and gallic acid possess antibacterial activity against both Gram-negative and Gram-positive bacteria, and ROS involvement has been demonstrated as an important contributor to the process [13, 21–23]. In this study, the combination of colistin with orientin, a catechol functional group bearing flavonoid, was evaluated against *K. pneumoniae* and *P. aeruginosa*. The observed high MIC values of orientin relative to colistin in the current study are not surprising because colistin is a conventional antibiotic that has been modified while orientin is a mere plant secondary metabolite that could be further modified to enhance its antibacterial effect. Nevertheless, judging by a previous submission [24] that MIC values ˃ 1000 *μ*g/mL should be avoided for crude extract and isolated compounds and that MIC range of phytocompounds should be between 100 and 1000 *μ*g/mL to be classified as antimicrobials [25], the results from this study regarding the MIC values can be regarded as remarkable for orientin with significant activity against the test organisms as its MIC values were less than 1000 *μ*g/mL.

Combination therapy allows the use of lower concentrations and therefore minimizes the advent of probable toxicity [26, 27]. The type of interaction that results from the combination of colistin and orientin against both *K. pneumoniae* and *P. aeruginosa* was synergistic as the FICI was 0.37 and 0.31, respectively. Based on the FICI values obtained in this study, orientin reduced the MIC value of colistin by three- and fourfold against *K. pneumoniae* and *P. aeruginosa*, respectively. This not only suggests that orientin enhanced the antibacterial activity of colistin, but also is indicative of its propensity to act on a different target other than the cell membrane like colistin [28]. In a previous study [8], morin and quercetin (flavonoids) enhanced the activity of ciprofloxacin and tetracycline against *Staphylococcus aureus* CECT 796, ciprofloxacin against *S. aureus* 1199B, and tetracycline against methicillin-resistant strains. This was said to be associated with the existence, amount, and degree of substitution of hydroxyl or methyl groups on the benzene ring. Hence, these properties could also have had a crucial role in the antibacterial activity of orientin in addition to its catechol group. In addition to checkerboard assay, time-kill kinetics further supported the synergistic interaction of colistin and orientin as both *K. pneumoniae* and *P. aeruginosa* cells were completely killed following treatment with their combination. These results suggest that the combination of colistin with orientin at the investigated concentrations was bactericidal against both isolates. However, the observed regrowth of *P. aeruginosa* cells after 24 h of treatment with either colistin or orientin alone could mean that colistin and orientin alone were bacteriostatic against *P. aeruginosa* at the investigated concentrations. This observation is in line with the report of Abreu et al. [8], where the regrowth of *S. aureus* SA1199B was observed after 8 h, following treatment with rutin, demonstrating the bacteriostatic effect of rutin at the investigated concentration.

The generation of ROS is regarded as one of the important aspects of antibiotics that induce oxidative stress in bacteria with subsequent contribution to its lethality [29]. Of the ROS generating pathways involved in bacterial lethality, the tricarboxylic acid (TCA) cycle plays a significant role [30]. According to Adam-Vizi and Chinopoulos [31], ROS, particularly superoxide anion radicals, are mainly produced in the mitochondria, and their production is associated with high NAD^+^/NADH levels. In this study, the high levels of NAD^+^/NADH ratio produced in the cotreatment relative to the single treatments particularly against *K. pneumoniae* could signify the involvement of oxidative stress in the killing of *K. pneumoniae* and *P. aeruginosa*. In addition to the increased NAD^+^/NADH ratio, the increased ADP/ATP ratio in *K. pneumoniae* and *P. aeruginosa* cells treated with colistin, orientin, and their combination may be indicative of ATP accumulation which could have resulted from the inhibition of energy-consuming processes such as phase 1 of glycolysis [30]. This inhibition could further inhibit the primary electron flow pathway (oxidation of NADH to NAD^+^ in the TCA cycle) together with the electron transport chain, and this could be transferred to oxygen molecules by side reactions to produce ROS as earlier reported [32]. Previous studies have implicated and documented the elevation of ROS, particularly superoxide anion radical, as one of the common mechanisms for bactericidal antimicrobials [32, 33]. The elevation of superoxide anion radical in the cells treated with either colistin, orientin, or their combination may be attributable to ROS generation. However, the elevation of superoxide anion radical was more observed in the cotreatment regimen. This could be due to the inhibition of electron transport chain activities, and this observation is consistent with the study of Ajiboye et al. [34], where only the cotreatment of colistin with phenolic acids increased the generation of superoxide anion radicals against both the wild type and mutant strains of *A. baumannii*.

A major issue associated with combination therapy is drug-drug interaction (DDI) which could result in serious harm to patients and even lead to death [35]. The CYP3A4 is one of the most vital isoenzymes belonging to the P450 family and is responsible for metabolism of several (> 60%) drugs, hence potentiating a crucial biological and medicinal application [36, 37]. Studies have revealed a higher risk of adverse effects and negative impact on the efficacy of coadministered drugs under influence of CYP3A4 [38, 39]. Thus, assessing the probable interactions between therapeutic agents and CYP3A4 is imperative to their application. According to Tallei [40], the formation of hydrogen bonds is one of the indices that could be used to establish the pattern of interactions between a ligand and a suitable receptor, which will, in turn, dictate the biological properties of the resulting complex. In this study, molecular docking against CYP3A4 revealed that the combination of colistin and orientin will not result in DDI when coadministered. This was evident from the observation that the number of hydrogen bonds formed in ketoconazole and rifampicin complexes was less than 5 contrary to those of colistin and orientin. Additionally, the observed hydrogen bonds with Ile 443, Gly 444, Arg 105, and Arg 212 at the binding pockets of CYP3A4 with either ketoconazole or rifampicin, which were absent in complexes with orientin and colistin, could be another good reason why neither colistin nor orientin is an inducer or an inhibitor of CYP3A4. This was further corroborated by the SwissADME prediction, thus allying the fear of DDI in the event of coadministration of colistin and orientin.

## 5. Conclusion

The emergence of antibiotic-resistant Gram-negative bacteria has continued to prompt the need for alternative strategies to overcome this menace. Combination therapy is one of the strategies that are gaining much interest as it addresses issues relating to resistance, while providing broad-spectrum antibacterial activity with reduced toxicological concerns. In this study, it was demonstrated that orientin potentiates colistin in the killing of *K. pneumoniae* and *P. aeruginosa* through the reduced MIC of colistin from 16 to 2 *μ*g/mL against *K. pneumoniae* and from 64 to 4 *μ*g/mL against *P. aeruginosa*. Furthermore, the increased level of NAD^+^/NADH and ADP/ATP ratios coupled with the generation of superoxide anion radicals revealed that the cotreatment of colistin and orientin induced oxidative stress, in a manner that enhanced bacterial lethality. Even though orientin acted synergistically with colistin, the exact mechanism through which orientin does this is still not clearly known. Hence, studies on information about the exact target and mechanism of antibacterial action of orientin are imperative and highly recommended.

## Figures and Tables

**Table 1:** Antibacterial activities of colistin and orientin and their friction inhibitory concentration index.

Test isolates	Zone of inhibition (mm)		MIC (*μ*g/mL)		MBC (*μ*g/mL)		FICI	Interactions
	Colistin	Orientin	Colistin	Orientin	Colistin	Orientin		
*K. pneumoniae*	23	25	16	64	128	512	0.37	Synergistic
*P. aeruginosa*	18	11	64	256	256	1024	0.31	Synergistic

MIC: minimum inhibitory concentration, MBC: minimum bactericidal concentration, and FICI: friction inhibitory concentration index.

**Table 2:** Interactions and binding energies of the compounds and standards against CYP3A4 protein.

Protein	Compounds	Binding energy score (kcal/mol)	No. of hydrogen bonds	Hydrogen bonds interactions	SwissADME remarks	
					Inhibitor	Inducer
CYP3A4	Colistin	−8.0	10	Cys 442, Ala 305, Pro 434, Thr 433, Arg 372, Ala 370, Gly 481, Phe 213, Ser 119	No	No
	Orientin	−9.0	5	Ala 370, Arg 106, Glu 374, Arg 105	No	No
	Ketoconazole	−9.5	2	Ile 443, Gly 444	Yes	No
	Rifampicin	−7.7	3	Arg 105, Arg 212	No	Yes

## Data Availability

The data used to support the findings of this study are included within the article.

## Supplementary Materials

Supplementary Materials Additional data for the results of the antibacterial activities of colistin and orientin as well as those of the molecular docking are presented in the supplementary file (Figures S1–S5).
