# Supplementary material for: Orientin Enhances Colistin-Mediated Bacterial Lethality through Oxidative Stress Involvement
Source: Evid Based Complement Alternat Med. 2022 May 9;2022:3809232. doi: 10.1155/2022/3809232 (PMC9110166; doi:10.1155/2022/3809232)
Supplement: Supplementary Materials — Additional data for the results of the antibacterial activities of colistin and orientin as well as those of the molecular docking are presented in the supplementary file (Figures S1–S5). [file 3809232.f1.docx]

**Supplementary figures**

**A B**


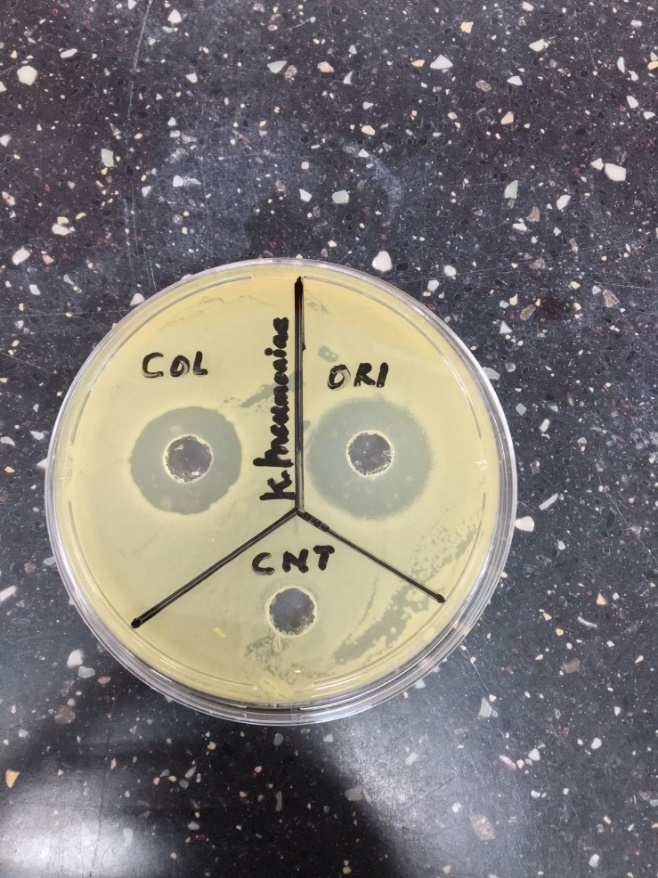

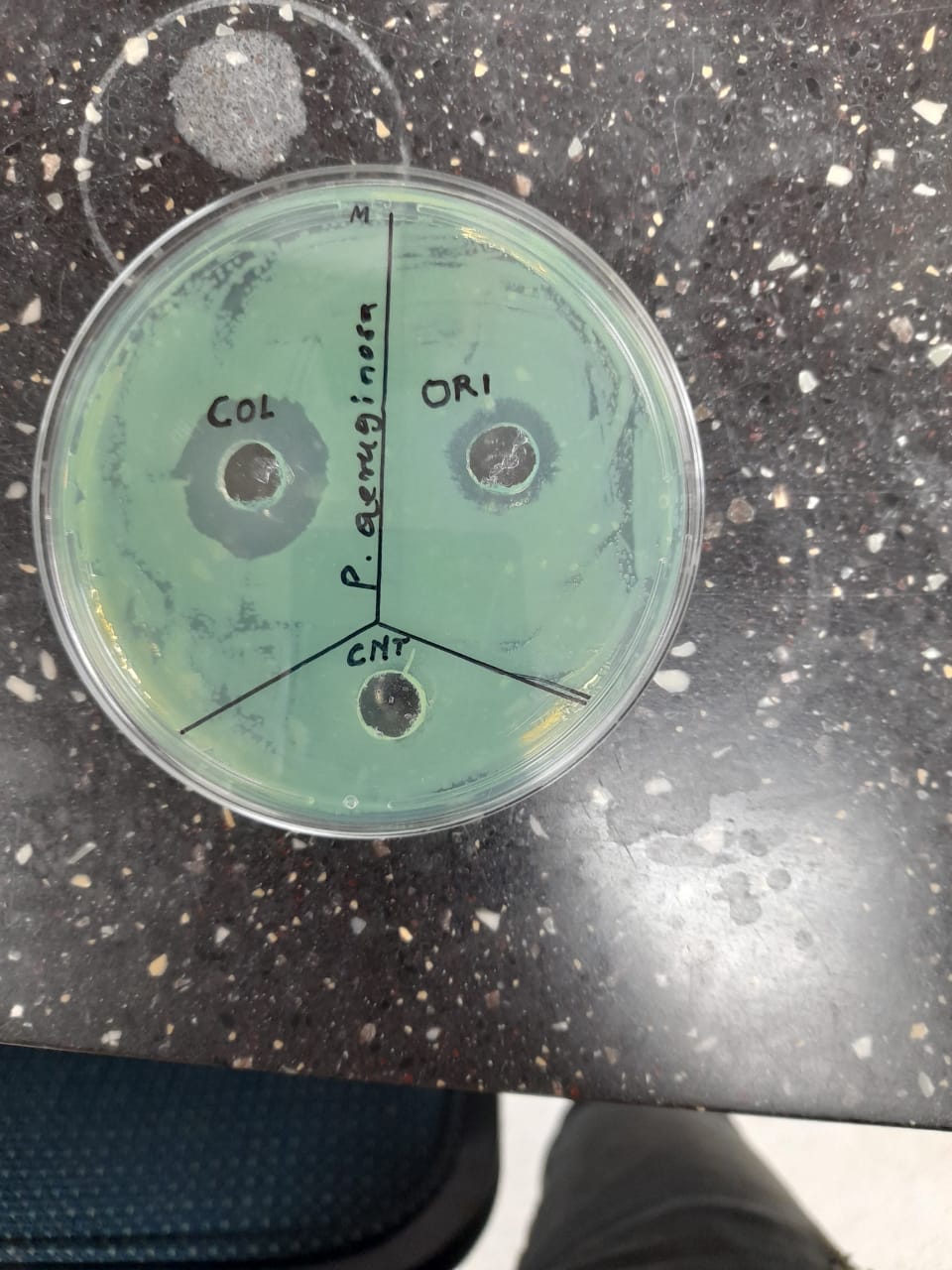


Figure S1. Zones of inhibition of colistin, orientin, and control (sterile distilled water) against (a) K. pneumonia and (b) P. aeruginosa. COL: Colistin, ORI: Orientin and CNT: Control.

**A**


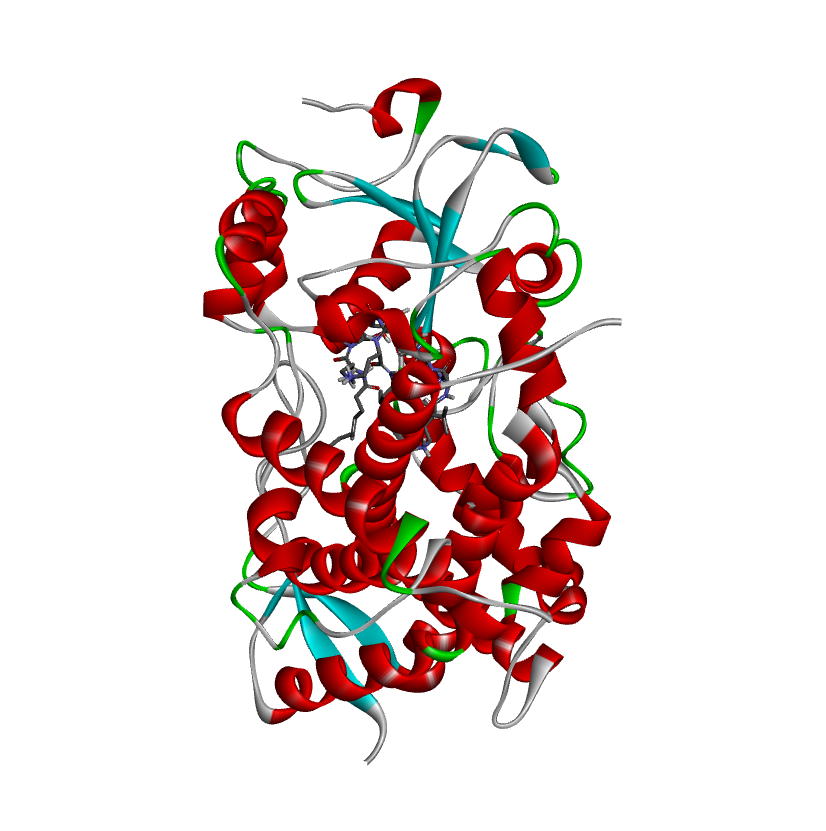


**B**


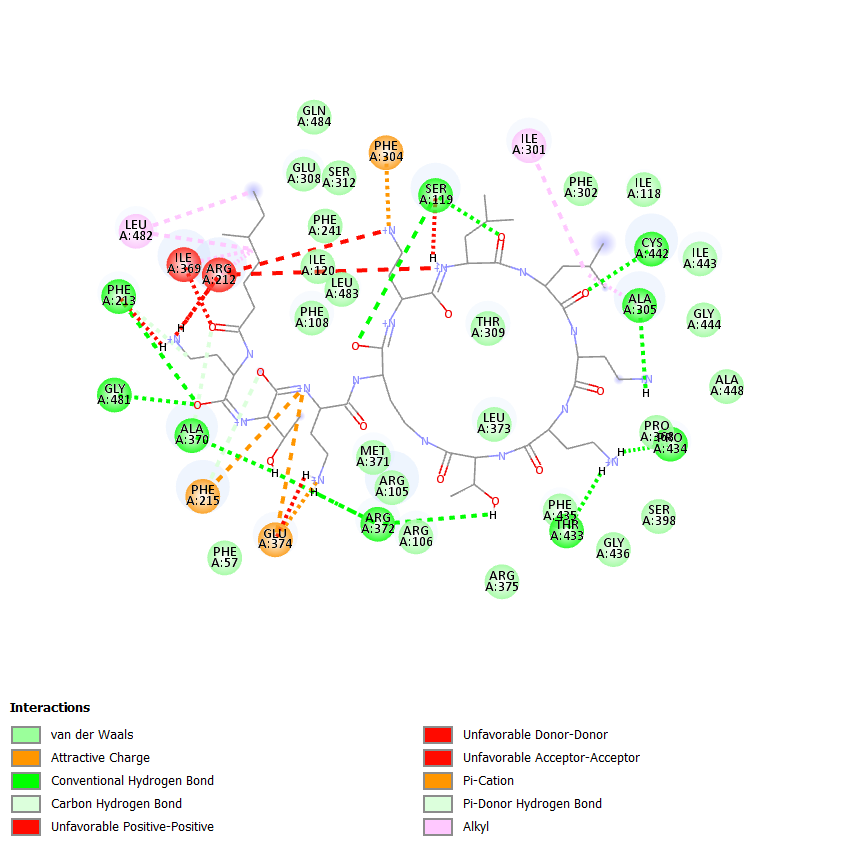


Figure S2. (A) 3D and (B) 2D interaction plots of colistin with CYP3A4

**A**


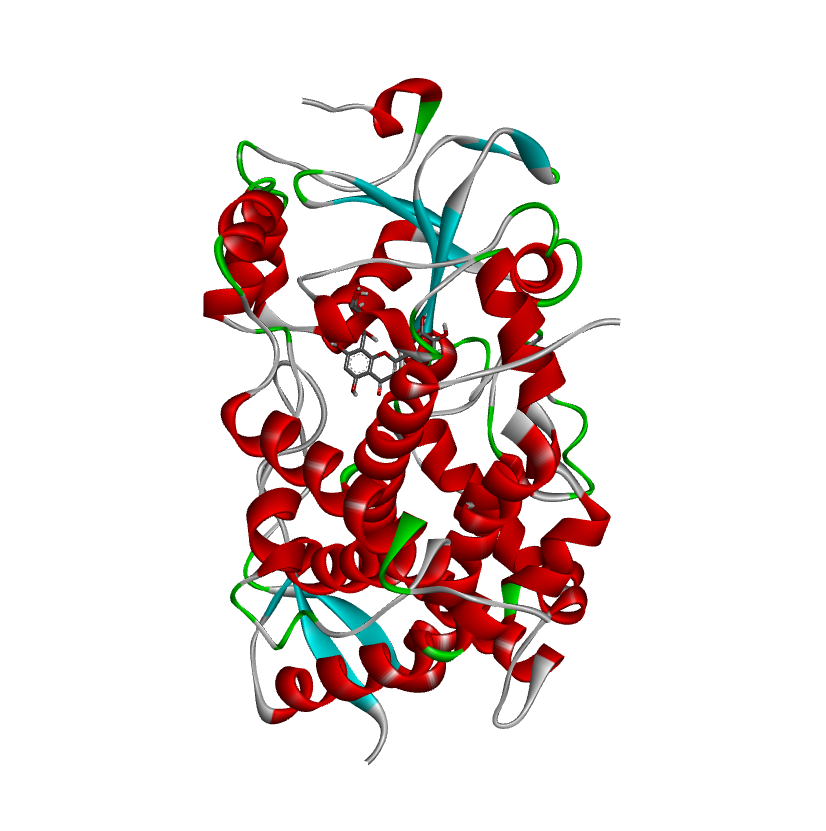


**B**


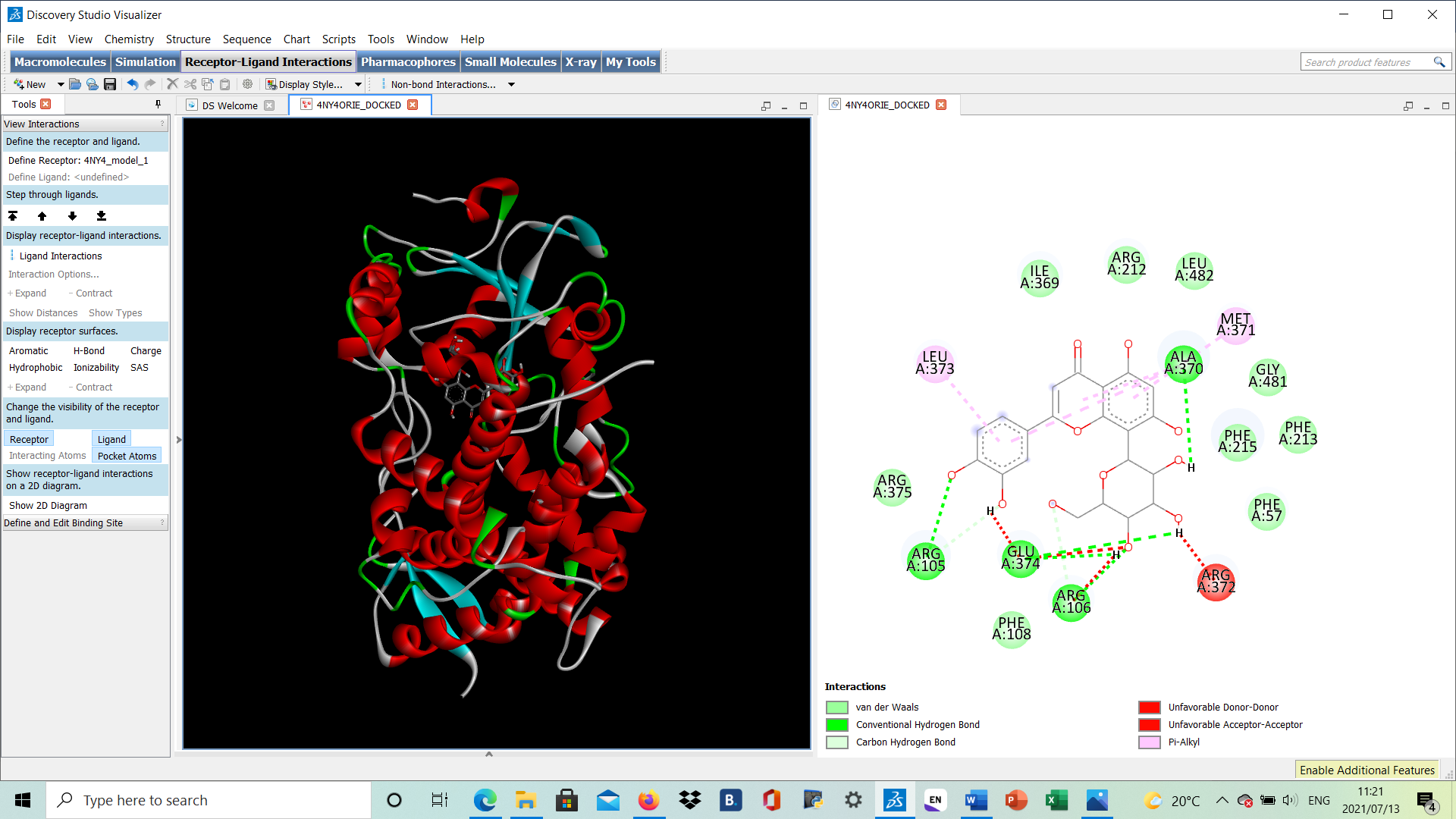


Figure S3. (A) 3D and (B) 2D interaction plots of orientin with CYP3A4

**A**


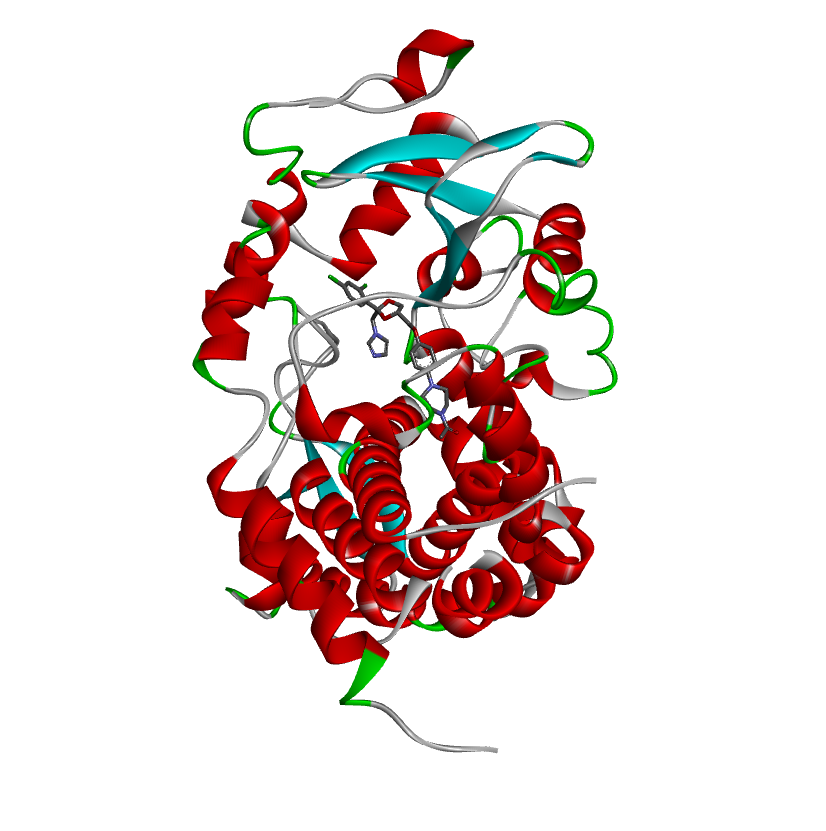


**B**


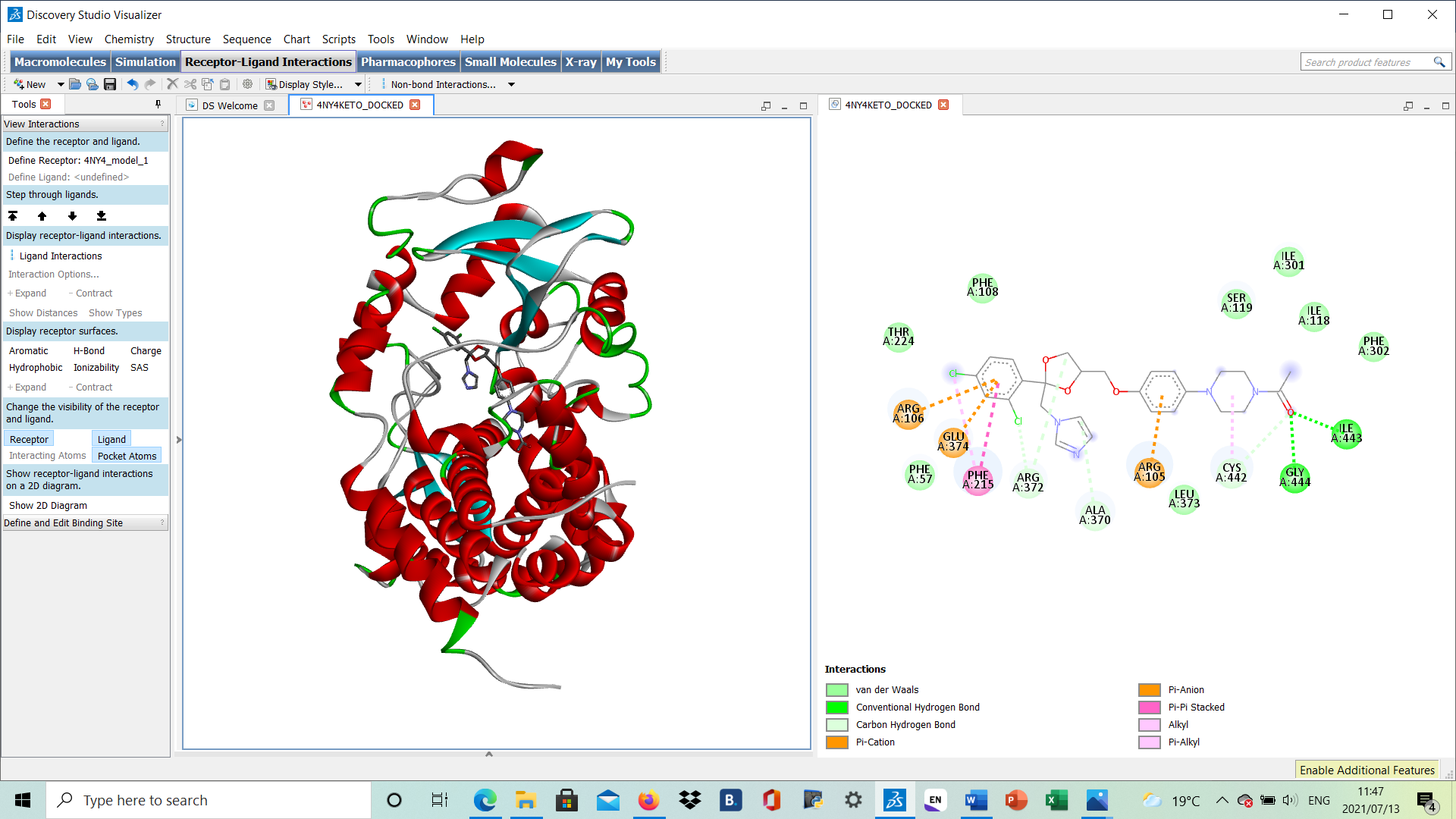


Figure S4. (A) 3D and (B) 2D interaction plots of ketoconazole with CYP3A4

**A**


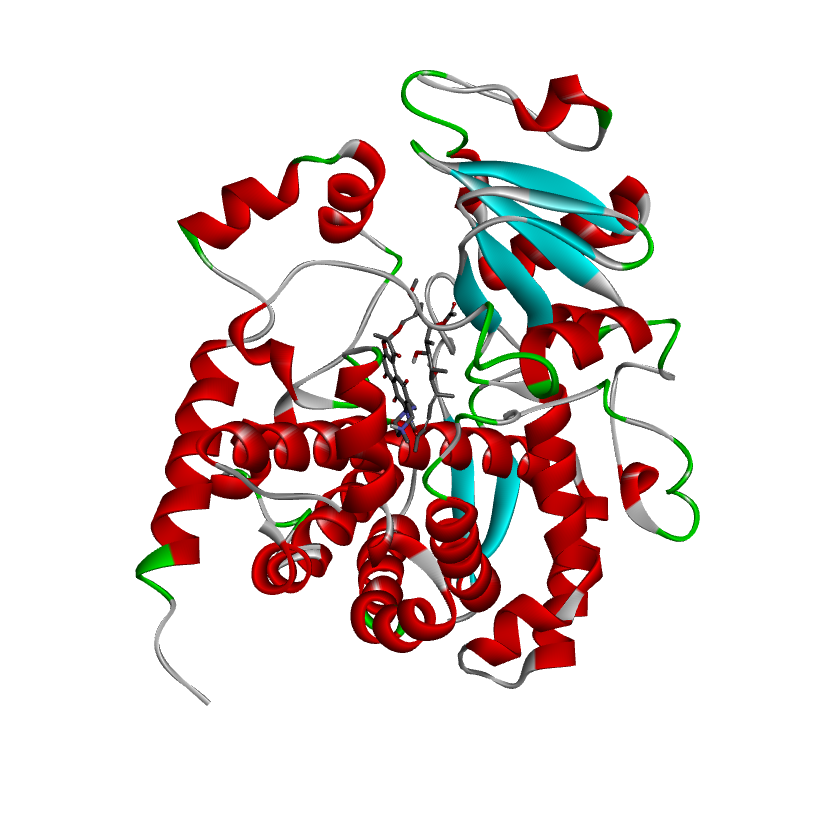


**B**


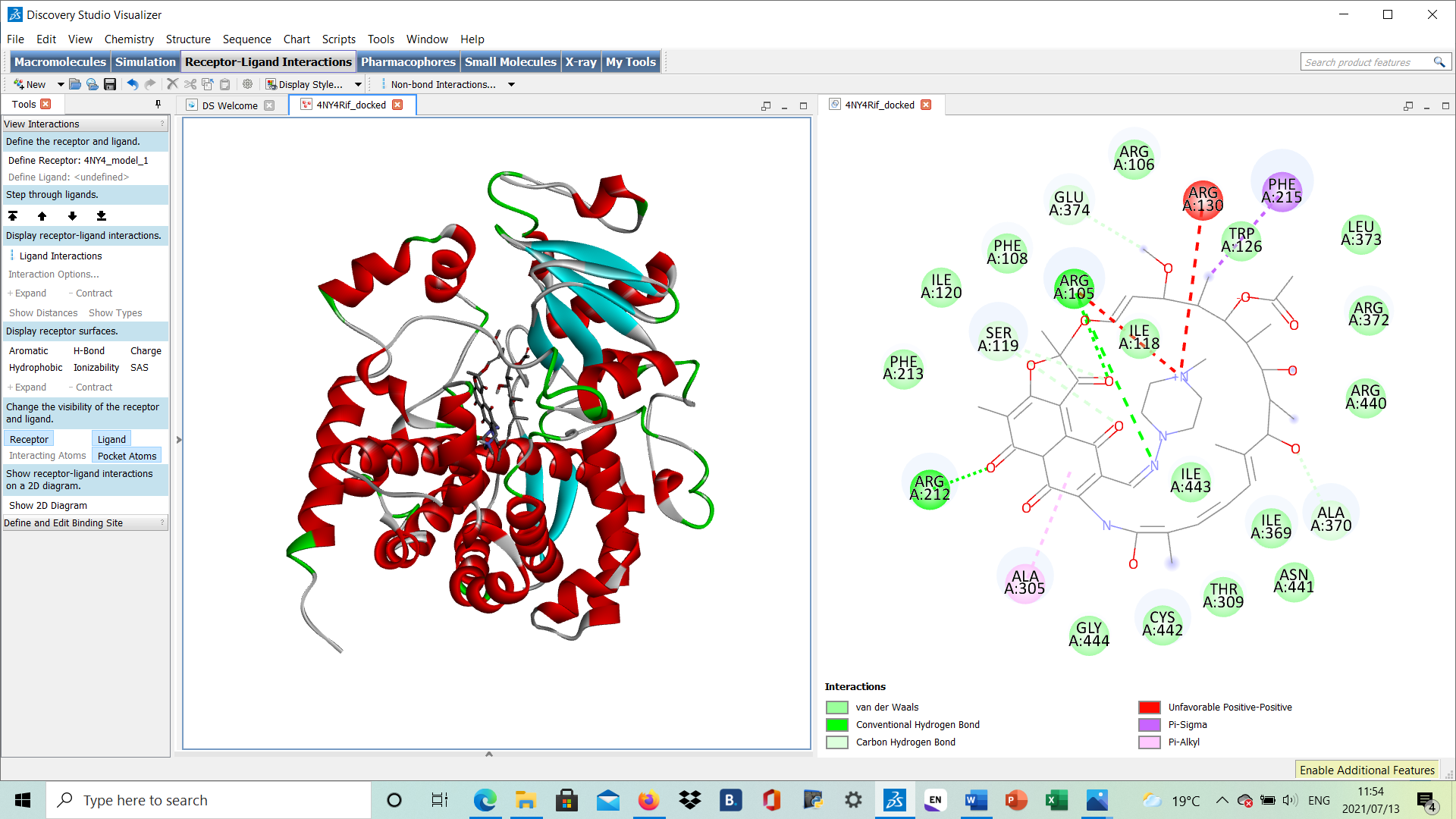


Figure S5. (A) 3D and (B) 2D interaction plots of rifampicin with CYP3A4
